# Genetically Modified Plants: Public and Scientific Perceptions

**DOI:** 10.5402/2013/820671

**Published:** 2013-03-07

**Authors:** Smita Rastogi Verma

**Affiliations:** Department of Biotechnology, Delhi Technological University, Delhi 110042, India

## Abstract

The potential of genetically modified plants to meet the requirements of growing population is not being recognized at present. This is a consequence of concerns raised by the public and the critics about their applications and release into the environment. These include effect on human health and environment, biosafety, world trade monopolies, trustworthiness of public institutions, integrity of regulatory agencies, loss of individual choice, and ethics as well as skepticism about the real potential of the genetically modified plants, and so on. Such concerns are enormous and prevalent even today. However, it should be acknowledged that most of them are not specific for genetically modified plants, and the public should not forget that the conventionally bred plants consumed by them are also associated with similar risks where no information about the gene(s) transfer is available. Moreover, most of the concerns are hypothetical and lack scientific background. Though a few concerns are still to be disproved, it is viewed that, with proper management, these genetically modified plants have immense potential for the betterment of mankind. In the present paper, an overview of the raised concerns and wherever possible reasons assigned to explain their intensity or unsuitability are reviewed.

## 1. Introduction

Genetically modified (GM) plants, also called transgenic plants, are designed to acquire useful quality attributes such as insect resistance, herbicide tolerance, abiotic stress tolerance, disease resistance, high nutritional quality, high yield potential, delayed ripening, enhanced ornamental value, male sterility, and production of edible vaccines. Another major goal for raising the GM plants is their application as bioreactors for the production of nutraceuticals, therapeutic agents, antigens, monoclonal antibody fragments biopolymers, and so forth [1]. Thus, GM plants can potentially affect many aspects of modern society, including agricultural production and medical treatment. Despite these potential applications, the use of GM plants for human welfare has been restricted owing to various concerns raised by the public and the critics. These concerns are divided into different categories, namely, health, nutritional, environmental, ecological, socioeconomic, and ethical concerns [2–25]. These concerns include those arising due to properties of GM plants themselves, those resulting from the spread of the transgenes to other organisms, and also those resulting from their release into the environment. Such concerns have led to the withdrawal of commercialization of *Bt* cotton and *Bt* brinjal in India. The campaign against GM plants was fueled by the instances of transgenic potatoes reported to be deleterious to rats, contamination of commercial corn products with unapproved StarLink and killing of monarch butterfly by *Bt* corn pollen [26–28]. Furthermore, the nongovernmental organizations (NGOs) such as Gene Campaign, Center for Sustainable Agriculture, Research Foundation for Science, Technology and Ecology, Greenpeace, and Friends of the Earth have also raised concerns related to genetic manipulation of plants [28, 29]. The regulators, activists, media personnel and scientific journals have been undiscriminating and overly tolerant of the misrepresentations and distortions of anti-GM activists [30, 31]. There are not even scientific explanations for some of the concerns, but today the amount of misinformation is such that it has become difficult to separate truth from public perception about the GM plants. The biotechnology scientists, however, believe that GM plants should be given public acceptance because most of the concerns are not specific for GM plants and can exist for non-GM plants as well. In the present paper, a review of public perspectives regarding GM plants and their disapproval on the basis of scientific background is presented. 

## 2. Concerns Related to Health and Nutritional Status 

In case the products of GM plants are to be consumed by humans and animals, there is always a fear and risk in the society that these plants may create health problems or may lead to the development of newer microbial strains that may be pathogenic. Further, the plants themselves may be susceptible to such risks. Public and critics are also skeptical about the nutritional content and quality of the GM plants. Such health and nutrition related concerns and their negation by scientists are described in this section.

### 2.1. Susceptibility to Allergens

One of the major distressing problems with nontraditional proteins in GM foods is the risk of introducing allergens (usually glycoproteins) into the food supply of humans and animals. The public is concerned about the nature of these new food proteins as their allergenic or nonallergenic qualities are unknown [32]. Allergenicity has been demonstrated in transgenic soybeans due to the transfer of a major food allergen from Brazil nuts [33]. On the other hand, the scientists believe that the food allergens are found only in a few defined sources (peanut and other grain legumes, shellfish, tree nuts, etc.), and hence, only a dozen foods may produce allergic reactions. Moreover, allergenicity occurs when these food allergens are present in large proportions in the food and the individuals are sensitized to them over time to cause any adverse effects. Thus, it is highly unlikely for new allergens to be introduced into the food supply from GM plants.

### 2.2. Transfer of Antibiotic Resistance Gene to Microbes and Reduced Efficacy of Antibiotic Therapy

Public is also concerned about the potential risks associated with gene transfer from plants to microbes. It is speculated that the consumption of GM foods containing antibiotic resistance marker gene (e.g., *Npt* II gene encoding neomycin phosphotransferase for resistance to kanamycin and neomycin or *Amp* ^r^ gene encoding *β*-lactamase for resistance to ampicillin) by humans and animals may lead to transfer of these genes from GM food to microflora in the gut of humans and animals or to the pathogens in the environment transforming them into strains that are resistant to antibiotic therapy [32]. The transfer of antibiotic resistance gene to unrelated microorganisms such as *Aspergillus niger* has also been demonstrated [34]. Biotechnology scientists, however, are of the opinion that the *Npt* II gene used to develop GM plants currently in the market is safe for use because there is no evidence of allergenicity or toxicity related to it. Moreover, humans are also susceptible to consuming several kanamycin resistant bacteria that occur naturally in the environment. Human gut is reported to contain 10^12^ kanamycin-resistant bacteria and by consuming a tomato harboring *Npt* II gene, the increase in frequency of kanamycin-resistant bacteria in the gut amounts only to 10^−6^%. Furthermore, acid conditions prevalent in stomach or rumen inactivate or degrade the encoded enzyme, neomycin phosphotransferase II. Also neomycin phosphotransferase II requires ATP for its activity, which is present in extremely low concentrations in the gut. Regarding the use of *Amp* ^r^ gene for selection of bacterial recombinants, it is not transferred to plants. Moreover, the *Amp* ^r^ gene is considered safe because it does not encode for any product in plants. The *Npt* II and *Amp* ^r^ genes have been declared safe to use in GM plants [35–37]. The public is, however, reluctant to accept this fact. Looking to the views of public, scientists have also developed nonresistance based selectable marker genes such as green fluorescent protein encoding gene (*Gfp*) and *β*-glucuronidase gene (*Uid* A) [38–40]. Besides, intron-containing *Npt* II gene has also been assessed as an efficient selectable marker in plant transformation [41]. Due to insertion of intron in the *Npt* II gene, the theoretical risk of gene flow from GM plants to enteric bacteria is eliminated. Strategies for the removal of antibiotic resistance genes have also been devised [42]. One such strategy is the cloning of selectable marker gene and the transgene on two separate transfer DNA (T-DNA) molecules in a single plasmid or on two separate plasmids that are contained in one or more *Agrobacterium tumefaciens* strains used for plant transformation. The transgene and selectable marker gene are, thus, inserted at the loci, which should recombine at reasonably high frequencies so that the transgene can be segregated from the selectable marker gene in the next generation [43, 44]. Second strategy to eliminate the selectable marker gene is to flank it with direct repeats of recognition sites for a site-specific recombinase so that the marker gene can be easily excised from the plant genome by recombinase-mediated site-specific recombination. Examples included in this category are the Cre/*lox* recombination system of bacteriophage P1, Flp/*frt* recombination system of yeast 2 *μ*m plasmid and R/*Rs* system of *Zygosaccharomyces rouxii*. A common feature of these systems is that the first round of transformation produces transgenic plants with the selection marker between two directly oriented recognition sites for the respective recombinase. After expression of recombinase, either by crossing in plants expressing the enzyme, by transient expression via second transformation, or by the use of an inducible promoter, the recombinase reaction is initiated resulting in marker-free transgenic plants [45–50]. Marker gene may also be eliminated by placing it on a transposable element resulting in its loss after transposition [39, 51]. The transgene by itself may be mobile and the activation of transposase allows the relocation of the desired transgene to a new chromosomal position. Genetic crosses and/or segregation may dissociate the two transgenes [52]. Another novel strategy for the production of marker-free GM plants involves DNA deletion based on intrachromosomal homologous recombination between two homologous sequences, for example, by incorporating *att* sequence of *λ* bacteriophage [53].

### 2.3. Development of New-Line Microbial Strains

The third health risk is related to the ability of GM plants to create new toxic organisms. It is speculated that some nonpest microbial strains may acquire pathogenic trait by gene flow from GM plants [32]. The risk can also be a new host being infected by a virus or recombining to form a more deadly virulent virus [54, 55]. Some plant pathologists also hypothesize that development of virus-resistant plants may allow viruses to infect new hosts through transencapsidation. Virus-resistant plants may also lead to the creation of new viruses through an exchange of genetic material or recombination between RNA virus genomes. Another matter of concern is that a small fraction of the DNA released from GM plants into soil may bind to the clay particles and hence protected from degradation. It is speculated that the soil bacteria may undergo transformation with the exogenous DNA of GM plant [56]. This is, however, a rare possibility as the amount of DNA derived from GM plants as a proportion of the total DNA in the soil is likely to be very small, even if such plants are grown on a commercial scale [57]. Moreover, the longevity of DNA in soil depends on various factors, including soil type and the presence of deoxyribonucleases in soil [11, 58–60]. Laboratory microcosm experiments have shown all but 0.1% of the target DNA from transgenic tobacco plants gets degraded within 40 days [56, 57].

### 2.4. Skepticism about Nutritional Status

Critics of GM crops have raised various concerns about the potential of golden rice to combat vitamin A deficiency (VAD). The primary concern amongst these is the presence of insufficient vitamin A in golden rice. There are still doubts about the speed of degradation of vitamin A after harvesting the plant and the amount of vitamin A left after cooking [61]. Vandana Shiva, an Indian anti-GMO activist, has criticized golden rice by saying “the golden rice is a hoax,” “golden rice is a blind approach for blindness control,” and “golden rice is just a recipe for creating hunger and malnutrition” [62–64]. She argues that the golden rice fails to pass the vitamin A need test and is incapable of removing VAD. It is calculated that one serving contains 30 g of rice on dry weight basis and golden rice can provide only 9.9 *μ*g of vitamin A, that is, only 1.32% of the required daily allowance (RDA) of 750 *μ*g. Even with the daily consumption of 100 g golden rice, only 4.4% of the required daily allowance will be met. Thus, an adult has to consume 2 kg 272 g of golden rice per day to complete his daily requirements of vitamin A. She is also of the view that, besides creating VAD, golden rice will also create deficiency in other micronutrients and nutrients. This is because the raw milled rice has a low content of fat (0.5 g/100 g), which is necessary for vitamin A uptake, low content of protein (6.8 g/100 g), which is required as a carrier molecule, and low content of iron (0.7 g/100 g), which is required for the conversion of *β*-carotene to vitamin A. Friends of the Earth, Greenpeace, and Vandana Shiva further emphasize that there is no need of golden rice to combat VAD as superior alternatives such as sweet potato, green leafy vegetables, coriander, amaranth, carrot, pumpkin, mango, jackfruit exist in nature [62–65]. It is reported that certain underutilized plants also exhibit far more nutritional value (vitamin A and other nutrients) than golden rice, for example, a combination of rice and leaves of *Moringa* (drumstick) tree, a native to India [62–64]. Similarly, in contrast to rice, amaranth grain contains forty times more calcium, four times iron, and twice as much protein. The ragi millet, grown in India, has thirty five times more calcium than rice, twice as much iron, and five times more minerals [66]. It is opined that golden rice is not capable of increasing the production of *β*-carotene. Even if the target of 33.3 *μ*g of vitamin A in 100 g of rice is achieved, it will be only 2.8% of *β*-carotene that can be obtained from amaranth leaves, 2.4% as that obtained from coriander leaves, curry leaves, and drumstick leaves [62–64]. Thus, a far more efficient route to removing VAD is biodiversity conservation and propagation of naturally occurring vitamin A rich plants (wild-type or underutilized) in agriculture and diets. Even the World Bank has admitted that rediscovering and use of local plants and conservation of vitamin A rich green leafy vegetables and fruits have dramatically reduced VAD threatened children over the past 20 years in very cheap and efficient ways. It is also speculated that the cultivation of golden rice will lead to major water scarcity since it is a water intensive crop and displaces water prudent sources of vitamin A. The scientists, on the other hand, believe that the traditional breeding methods have been unsuccessful in producing crops containing a high vitamin A concentration and most national authorities rely on expensive and complicated supplementation programs to address the problem. They also believe that a varied diet is beyond the means of many of the poor and they have to rely on one or few foods to provide complete nutrition, for example, rice. Thus, golden rice may be a useful tool to help treat the problem of VAD in young children living in the tropics. They also emphasize that the critics are ignoring the fact that VAD disorders result from a deficiency of vitamin A and not its complete absence in the diet and the VAD individuals lack only 10%–50% of their daily requirements. Hence, any additional contribution toward daily requirements would be useful. In 2005, a team of researchers at Syngenta have produced a variety of golden rice, called “Golden rice 2,” which produces twenty-three times more carotenoids than golden rice (up to 37*μ*g/g) and preferentially accumulates *β*-carotene (up to 31*μ*g/g of the 37*μ*g/g of carotenoids) [67]. The Rockefeller Foundation emphasized that the new strains of golden rice contain substantially higher levels of *β*-carotene than the early versions on which the opponents based their calculations. In order to meet the RDA, 144 g of the most high-yielding golden rice strains would have to be eaten. 

## 3. Environmental and Ecological Concerns

Large-scale cultivation of GM plants expressing viral and bacterial genes and their release into the environment is considered to be a threat and called as “genetic pollution” by the critics [68–76]. The risk of a transgene spreading in the environment is related to the likelihood for out-crossing, horizontal gene transfer, and the phenotype imparted by the gene [72]. Debates about the commercial introduction of GM plants in some parts of the world have led to questions about their potential impact on the environment unless necessary safeguards are taken into account [77]. Various concerns that have arisen due to the application and release of GM plants into the environment are given in this section. It should, however, be acknowledged that agriculture inevitably has an impact on the environment and these concerns are not specific for GM plants. 

### 3.1. Transgene Escape to Wild-Type Plants

There is a potential risk that the GM plants may hybridize (or cross-breed) with sexually compatible wild-type species [71, 78–82]. This genetic exchange is possible due to wind pollination, biotic pollination or seed dispersal. This may have an impact on the environment through the production of hybrids and their progeny. In an example, virus-resistant squash commercialized in 1994 was demonstrated to transfer its virus resistance gene to wild squash (*Cucurbita pepo*), an agricultural weed native to the southern United States, thereby decreasing its value to squash breeders [83, 84]. On the other hand, it is significant to note that for an effective pollen transfer to occur, the GM plants must be close enough to the wild species, should flower at same time, and must be genetically compatible [78–81, 85]. Further, the risk of any gene transfer to related weedy species through pollen has been eliminated by devising chloroplast transformation procedures [86, 87]. This is because, in many crop species, chloroplasts display only maternal inheritance.

### 3.2. Selective Advantage to GM Plants in Natural Environments and Generation of Superweeds

The concern of gene flow from GM plants to weedy relatives via pollination is quite intense [72, 88–91]. It is considered that the transfer of encoded characteristics to weed species could potentially give them a selective advantage, consequently leading to the generation of “superweeds.” Moreover, the newly introduced traits may make a plant, especially herbicide tolerant plant, more persistent or invasive (weedy) in agricultural habitats [92–101]. It is, however, pertinent to note that the risk of gene transfer to weeds is similar with both conventional and GM plants and is not contingent on how these genes have been introduced into plants. Such a risk of gene flow has always existed since the advent of modern plant breeding, even when there were no GM plants, and this can occur where possible. Several studies have demonstrated that tolerance to particular herbicide is often more likely to develop by evolution from within the weed gene pool rather than by gene flow from herbicide-tolerant plants [102, 103]. Nevertheless, the current scientific evidence indicates that the weediness arises from many different characters and that the addition of one gene is unlikely to cause a crop to become a weed. The transfer of novel genes from transgenics (or even conventionally bred plants) to weeds depends on the nature of the novel gene and the biology and ecology of the recipient weed species. The probability of successful out-crossing thus depends on sexual compatibility, physical proximity, distance of pollen movement both out of and into the GM plants, and ecology of recipient species [78–81]. Thus, only a few plants such as oilseed rape, barley, wheat, beans, and sugar beet can hybridize with weeds. For example, oilseed rape has been reported to hybridize with hoary mustard, wild radish, and other wild *Brassica* species [80, 104–106]. Furthermore, the transfer of herbicide tolerance gene is unlikely to confer any competitive advantage to hybrids outside agricultural areas. It is also comforting to recognize that there is no proven evidence of enhanced persistence or invasiveness of GM plants and no major superweeds have developed so far. 

### 3.3. Effect on Nutritional Composition of Plants

It is also speculated that the nutritional composition of GM products may be affected in GM plants. Another concern is that the transgenes from animals (obtained from fishes, mouse, human, and microbes) introduced into GM plant for molecular farming may pose a risk of changing the fundamental nature of vegetables. In a study, it was reported that as compared to non-GM soybean, GM plants exhibited lower levels of isoflavones [107]. This finding also raised a doubt on the regulatory system for the release of the GM plants. However, later it was found that the concentration of isoflavone in GM soybean was within the normal range [108]. 

### 3.4. Mixing Genes from Unrelated Species (Interbreeding)

The public is worried about the risk that the GM plants can spread through nature and interbreed with natural organisms, thereby contaminating “non-GM” environments. This would in turn affect the future generations in an unforeseeable and uncontrollable way [72]. Such worries, however, ignore the history of plant breeding and the existing overwhelming sequence similarity of genes across kingdoms. 

### 3.5. Development of Tolerance to Target Herbicide

It is viewed that the repeated use of the same herbicide in the same area to remove weeds amongst genetically modified herbicide-resistant crops (HRCs) (tolerant to single herbicide) will exacerbate the problem of herbicide-tolerant weeds [72]. Another matter of concern relates to the plants carrying different herbicide tolerance genes to become multiply tolerant to several herbicides by pollination between adjacent plants [109]. In several closely studied examples in Canada, farmers have detected oilseed rape plants tolerant to three different herbicides (note that two were acquired from GM plants and the third possibly from conventional breeding) [110]. The development of multiple tolerances in “volunteer” crop plants (from seeds remaining viable in agricultural soil) may also exert an impact on the environment by necessitating the use of less environment-friendly and possibly outdated herbicides by the farmers. On the other hand, the proponents believe that herbicide resistance develops due to excessive application of herbicide and is not exclusively associated with gene transfer from genetically modified HRCs. Thus, the pressure on weeds to evolve resistant biotypes has been reported to be pronounced with the excessive application of herbicides such as glyphosate, sulphonylureas, and imidazolinones. 

### 3.6. Sustainable Resistance in Insect Pests

It is possible that the widespread use of disease-resistant GM plants may lead to the evolution of several insect pests that are resistant to pesticides [111–115]. For example, *Bt* crops may develop resistance to *Bt* biopesticide, a permitted biopesticide successfully used by organic farmers in the integrated pest management (IPM) programs. There is to date no reported evidence of insect resistance to *Bt* crops under field conditions although *Bt* resistant insects (e.g., cotton budworm and bollworm) have been observed in areas where *Bt* biopesticides are sprayed on crops [116]. It has been a matter of concern that the development of such resistance may lead to the loss of the potential of the *Bt* biopesticide, which may in turn make it necessary for organic farmers to resort to less environmentally acceptable chemical pesticides. Therefore, proper resistance management strategies along with this comparatively newer technology are imperative. The most widely used is the ‘high-dose refuge' strategy designed to prevent or delay the emergence of Bt toxin-resistant insects. Scientists are of the opinion that this strategy should be followed without fail, as the rate of noncompliance can increase the risk of plant resistance breakdown.

### 3.7. Harm to Nontarget Organisms

Nontarget effect, that is, undesirable effect of a novel gene (usually conferring pest or disease resistance) on “friendly” organisms in the environment, is another concern related to GM plants [117]. As many nontarget microbes harbor on plant surfaces or some insects harbor on flowers, it becomes quite challenging to target the insect resistance gene product to appropriate plant tissues and hence kill pests without exerting any adverse effect on friendly organisms such as pollinators and biological control agents. This is particularly difficult where the benign or beneficial organism is related and physiologically similar to the pest to be targeted. One of the most significant studies of nontarget impacts of GM plants has been the killing of monarch butterfly in the United States by Bt insecticidal proteins [9, 17, 27, 118–122]. It should, however, be noted that the pesticidal sprays used on *Bt* or non-*Bt* corn may be more harmful to the monarch butterfly as compared to *Bt* corn pollen [117]. Thus, in evaluating the use of *Bt* crops and the possible environmental damage caused, it is important to take into account the environmental damage caused by the use of pesticides in agriculture generally. It is argued that millions of birds and billions of insects, both harmful and beneficial, are killed each year due to excessive use of pesticides. It is, however, suggested that the scale and pattern of use may mitigate the effects of *Bt* on nontarget populations [123]. Furthermore, when toxins are produced within plant tissues, nontarget organisms are exposed to a much lesser extent than with spray applications because only those organisms which feed on the plant tissues come into contact with the toxin.

Harmful effect of *Bt* toxin residues in the soil after harvest of the GM crop on soil invertebrates has been another matter of concern. An investigation of the effect of *Cry1Ab* released from the roots and crop residues on soil organisms revealed the presence of toxin in the guts and casts of tested earthworms. There was, however, no significant difference in their mortality or weight. Moreover, no difference in the total number of other soil organisms (including nematodes, protozoa, bacteria, and fungi) between the soil rhizosphere of *Bt* and non-*Bt* crops was detected [124].

### 3.8. Increased Use of Chemicals in Agriculture

On one hand, the transgenes conferring herbicide resistance have been criticized because these would maintain, if not promote, the use of herbicides and their attendant problems [125, 126]. Similarly, there is a concern that the insect-resistant and disease-resistant GM plants will increase the application of insecticides and pesticides, respectively. On the contrary, reports demonstrate that there is no significant change in the overall amount of herbicide use in the United States since the introduction of GM soybeans [127]. An analysis by soybean growers at the United States has shown that $7.2 millions of other herbicides were replaced by $5.4 millions of glyphosate [19]. This substitution, thus, resulted in the replacement of highly toxic and more persistent herbicides with that of glyphosate. Furthermore, it has been reported that herbicide-tolerant oilseed rape eliminates the use of >6,000 tons of herbicide in the growing season [128]. 

### 3.9. Loss of Biodiversity

The public has long been worried about the loss of plant biodiversity due to global industrialization, urbanization, and the popularity of conventionally-bred high-yielding varieties. It is speculated that the biodiversity will be further threatened due to the encouraging use of GM plants. This is because development of GM plants may favor monocultures, that is, plants of a single kind, which are best suitable for one or other conditions or produce one product [72, 98, 129]. Further, the transformation of more natural ecosystems into agricultural lands for planting GM plants is adding to this ecological instability. 

Another point of concern is the loss of weed diversity that may occur due to gene flow from HRCs to weeds [126]. It is argued that because the currently available HRCs confer tolerance to broad-spectrum herbicides such as glufosinate and glyphosate, their extensive use may shift the diversity of weeds in agricultural habitats. However, weeds exhibit considerable plasticity and adapt to a wide range of cultivation practices. Experience with conventional agriculture has shown that weed species composition varies within the same crop among different fields and at different times of year. Thus, weed population shifts are natural ecological phenomena in crop management and should not be viewed as exclusive to GM plants.

### 3.10. Unpredictable Gene Expression

It is speculated that the random gene insertion, transgene instability, and genomic disruption due to gene transfer may result in unpredictable gene expression. Such a risk is, however, unlikely to be unique to GM plants or of any significance considering our current knowledge of genomic flux in plants.

### 3.11. Alteration in Evolutionary Pattern

Plants adapt to the fluctuations in the environment through changing their genes and developing better races called “evolved races.” These mutations, however, occur at a very low frequency (i.e., one in about 10^9^/gene/generation). It is hypothesized that the cultivation of GM plants by the farmers at an increasing rate throughout the world may change the evolutionary pattern drastically [72]. Another concern is the evolution of non-GM plants through hybridization with GM plants.

### 3.12. Loss of Ecosystem in Marginal Lands

As new plants are introduced mainly to marginal lands, loss of natural ecosystems in these areas has also been a matter of concern. 

### 3.13. Contamination of Soil and Water

It is also sometimes argued that the widespread introduction of HRCs will increase the use of herbicides, which will in turn contribute to the contamination of soil and ground water. However, this is not the case. The cultivation of HRCs in the United States has been reported to facilitate zero-till agronomic system, which contributes to a reduction in soil erosion. The release of *Bt* toxin into the soil after harvest of *Bt* crops is also viewed as a risk factor associated with the cultivation of *Bt* crops [123, 124, 130]. It has been found that *Bt* toxins remain active in soil; however, it is not necessarily an environmental hazard because *Bt* toxins must be ingested and affect only selected groups of insects. Moreover, the potential leaching rate of *Bt* toxin is reduced due to its binding and adsorption on clay particles [131].

## 4. Socioeconomic and Ethical Concerns

As the GM plants are likely to affect the society, their application is also related to certain social and ethical concerns. Besides, evaluation of their cost effectiveness (production cost versus potential benefits) is also a matter of concern. It is pertinent to note here that most of these concerns pertain to developing countries. A list of various socioeconomical and ethical concerns is presented below.

### 4.1. Slow Progress Rate

Critics are skeptical about the ability of genetic manipulation to increase food production and project that there will only be about slight increase in crop yield during the next decades [132]. Some persons further question why after so many years of research genetic engineers have not produced any high-yielding crop variety. The answer, according to plant scientists, is that plant breeders using traditional breeding techniques may have largely exploited the genetic potential for increasing the share of photosynthate that goes into the seed. Others feel lack of funds for pursuing research in the area of genetic manipulation of plants. Furthermore, the public ignorance about the GM technology is the prime factor for its slow progress rate. 

### 4.2. Prevalence of the Western Agriculture, Monopoly of Transnational Companies, and Exploitation of the Poor

The public in developing countries were of the opinion that there is domination of majority of the biotechnology industry by transnational companies (TNCs) in the developed world the business of which is to generate profits [132]. One such example is that many HRCs raised by genetic manipulation belong to the group of key crops in Western agriculture. The “terminator gene technology” developed by TNCs was also criticized as the technology was considered as a step to build monopoly over transgenic seed production [133, 134]. An apprehension related to the application of this technology was its accidental transfer to other varieties and related species of a specific crop through cross-pollination resulting in large-scale sterility. It has also been argued that pollen from crops carrying terminator trait would infect the fields of farmers who either rejected or could not afford this technology. Further, the imposition of heavy fees for use of seeds will lead to loss of control of cooperatives by local farmers. It is further argued that greater privatization will increase both legal and financial barriers to use of varieties. Activist groups view golden rice not as a boon for the world's hungry population but as a public relations campaign for the biotech industry. Charles Margulis of the Greenpeace Genetic Engineering Campaign viewed that the industry has shamelessly used golden rice in an attempt of the developed nations to quell growing distrust of its experimental foods. One social concern about the development of GM plants raised by the Third World countries is that TNCs may disadvantage poor farmers in developing countries, for example, for the packaging of GM seeds [132]. The situation is made even more complex because the majority of the genetic resources and thus biodiversity on which genetic manipulation depends are found in developing countries. Thus, in order to sustain the Third World, the targets should also include other plants, and these countries should be helped in bypassing expensive and high input crop production and in moving their traditional agriculture toward low input sustainable practices. 

### 4.3. Loss of Foreign Income and Employment

Another apprehension regarding the application of GM plants is the loss of export market as their products get substituted by production of alternatives generated by genetic engineering in industrialized countries [132]. It is viewed that this will result in unemployment and loss of foreign income in the developing countries. Further, the large agricultural estates will be strengthened leading to dislocation of small-scale landholders and farmers. The requirement of labour for cultivation is also speculated to reduce. It is expected that the generation of genetically modified HRCs may reduce labour market in weeding and may also increase dependence on foreign imports of chemicals. 

### 4.4. Unaffordability by Poor

Vandana Shiva also argued about the problems with poverty and loss of biodiversity in food crops, which are aggravated by the corporate control of agriculture based on GM foods [64]. She also argued that food security and nutritional security should be secured by some lower-cost, accessible, and safer alternative to GM rice, for example, amaranth, *Moringa*, sweet potato, green leafy vegetables, and so forth. 

### 4.5. Intellectual Property Rights and Patents Issue

As genes extracted from ecosystems in developing nations are exploited for raising GM plants in the developed nations, it is quite possible for them to get the patents [132]. It has become a matter of concern because it will result in developing world farmers paying for the products that originated from their nation's own resources.

### 4.6. Ethical Issues

Certain groups of public, including religious bodies, find it very unethical or inhumane to introduce human or animal genes into plants [135]. For example, the transfer of animal genes such as *α*-interferon gene into plants is objectionable to the vegetarians. Such concern was one of the reasons due to which the concept of “edible vaccines” did not gain much impetus.

### 4.7. Labeling and Segregation of GM Foods

The public has always lived with food risks, but in the last few decades, they have become concerned about the contents in GM foods [135, 136]. Such concern was never there with the foods derived from classically bred plants. The proponents say that such a question is ridiculous because like GM plants the information regarding the contents has never been there with classically bred plants. Moreover, with GM plants, at least the source of new genetic material being introduced is known, and hence there is possibility of testing predictable and even many unpredictable effects. It is suggested that for GM foods to come in the market, a compromise among government, seed producers, farmers, and consumers may be practical. This involves the labeling of GM ingredients and segregation of GM plants and seeds from conventional ones. 

## 5. Conclusion

Genetic Engineering Approval Committee (GEAC) granted permission to Maharashtra Hybrid Seed Company (Mahyco) in 2002 for commercial cultivation of three cotton hybrids, namely, MECH-12 *Bt*, MECH-162 *Bt*, and MECH-184 *Bt* after several years of field trials [1, 137, 138]. These were developed by introgression of insect resistance from *Bt*-containing Cocker-312 (Event MON 531) developed by Monsanto Corporation, USA, into parental lines of Mahyco propriety hybrids. These transgenic cotton plants (*Bt* cotton) harbored crystal protein gene (*Cry1Ac*) from the soil bacterium *Bacillus thuringiensis* and were resistant to infestation by Lepidopteran insects. Similarly, *Bt* brinjal (Event EE 1), harboring *Cry1Ac* gene obtained from Monsanto, was developed by Mahyco by introgression into various local varieties by University of Agricultural Sciences, Dharwad and Tamil Nadu Agricultural University, Coimbatore, through plant breeding [139, 140]. In 2006, an expert committee examined the biosafety data presented by Mahyco and concluded that *Bt* brinjal was safe and equivalent to its non-*Bt* counterpart according to the provided data; however, these findings should be reconfirmed by further field trials and the benefits of *Bt* brinjal with respect to existing methods for pest management and pesticide reduction should be ascertained. A second expert committee examined the data from these trials and approved its commercialization in India in 2009. However, for both *Bt* cotton and *Bt* brinjal, the government of India applied a moratorium on their release upon outcry by some scientists, farmers, and anti-GM activists due to biosafety reasons [1, 137–140].

Thus, public opinion regarding the application and development of genetic engineering is likely to be an important factor influencing the future development of the technology and its subsequent application within the commercial sector. It is, therefore, recommended that scientific research aimed at risk analysis, prediction, and prevention, combined with adequate monitoring and stewardship, must be done so that negative impact from GM products, if any, may be kept to a minimum. In this direction, a combination of demographic data from existing non-GM populations, simulation modeling of transgene dispersal, and monitoring field releases may guide in the assessment of risks related to the release of GM plants into the environment. Further, it is viewed that case-by-case studies can help in solving the raised concerns. Besides, public should be well informed that most of their concerns are skeptical and GM plants have tremendous potential in solving the present problems.
